# Hypoxia-Induced Biosynthesis of the Extracellular Matrix Molecules, Perlecan and Fibronectin, Promotes the Growth of Pleomorphic Adenoma Cells In Vitro Models

**DOI:** 10.3390/biomedicines11112981

**Published:** 2023-11-06

**Authors:** Satoshi Maruyama, Manabu Yamazaki, Tatsuya Abé, Jun Cheng, Takashi Saku, Jun-ichi Tanuma

**Affiliations:** 1 Oral Pathology Section, Department of Surgical Pathology, Niigata University Hospital, 1-754 Asahimachi-dori, Chuo-ku, Niigata 951-8520, Japan; 2 Division of Oral Pathology, Department of Tissue Regeneration and Reconstruction, Faculty of Dentistry & Niigata University Graduate School of Medical and Dental Sciences, 2-5274 Gakkoucho-dori, Chuo-ku, Niigata 951-8514, Japanabet@dent.niigata-u.ac.jp (T.A.); tanuma@dent.niigata-u.ac.jp (J.-i.T.); 3 PCL Fukuoka Pathology Cytology Center, 4-11-32 Yoshizuka, Hakata-ku, Fukuoka 812-0041, Japan

**Keywords:** pleomorphic adenoma, hypoxia-inducible factor-1α (HIF-1α), extracellular matrix (ECM), heparan sulfate proteoglycan (perlecan), fibronectin (FN), proliferation, migration

## Abstract

Salivary pleomorphic adenoma is histopathologically characterized by its colorful stroma with myxoid, chondroid, and hyaline appearances, due to enhanced biosynthesis of extracellular matrix (ECM) molecules and poor vascularity. Thus, pleomorphic adenoma cells embedded in the stroma typically survive under hypoxic conditions. We determined the expression kinetics of ECM molecules, such as perlecan and fibronectin (FN), under hypoxia in SM-AP1 cells which are duct epithelial differentiated cells, and in SM-AP4 cells, which are myoepithelial differentiated cells, cloned from pleomorphic adenoma of the parotid gland. We investigated hypoxia-inducible factor-1α (HIF-1α)-inducing pathways through a variety of ECM molecules in association with their cellular proliferation and migration. We observed that hypoxic conditions with elevated HIF-1α protein levels induced increased expression of perlecan and FN in SM-AP cells than in controls. Moreover, perlecan and FN knockdown reduced the proliferation of SM-AP1 and SM-AP4 cells under hypoxia. Further, SM-AP1 cell migration was enhanced by both perlecan and FN knockdown, whereas SM-AP4 cell migration was increased by perlecan knockdown and inhibited by fibronectin knockdown. The results indicated that pleomorphic adenoma cells can survive under hypoxic conditions by promoting cell proliferation via enhanced synthesis of ECM molecules. Overall, ECM molecules may be a new anti-tumor target under hypoxic conditions.

## 1. Introduction

Pleomorphic adenoma is the most common type of benign salivary gland tumor, clinically characterized by slow-growing painless swelling. Histopathological analysis of pleomorphic adenoma revealed glandular and mesenchymal-like tumor cells embedded in various types of stroma produced by tumor cells themselves [1,2]. Although the tumor is usually well-encapsulated, occasional recurrences after surgery [2,3], and rare metastasis to carcinomas have been reported [1,2,4], which develop into secondary carcinomas called focal carcinoma [5] or carcinoma ex-pleomorphic adenoma, arising within the benign pleomorphic adenoma [6,7,8]. We had previously reported the establishment of SM-AP cell lines from primary cultures of pleomorphic adenomas that contain cells with proliferative potential in order to conduct experiments using pleomorphic adenoma cells with the above-mentioned characteristics [9].

Moreover, we had previously demonstrated that the capsule of pleomorphic adenomas is not always intact but is occasionally penetrated by tumor cell nests [10] and that its stromal architecture is characterized by hypo-vascularity, hypoxia, and abundant accumulation of extracellular matrix (ECM) molecules produced by tumor cells themselves [10]. The stroma of pleomorphic adenomas has histological variations including myxoid, fibrous, hyaline, and cartilaginous appearance, and rarely with osseous metaplasia [2]. The myxoid stroma is enriched in ECM molecules, such as the basement membrane-type heparan sulfate proteoglycan, perlecan [10], and fibronectin (FN) [11], especially in foci penetrating capsules, eventually resulting in recurrence [2,3,10]. In addition to the stroma of pleomorphic adenoma, the myxoid appearance due to perlecan deposition has also been demonstrated in the characteristic pseudocystic spaces of adenoid cystic carcinoma of the salivary gland [12], ameloblastoma [13], and oral squamous cell carcinoma (SCC) [14,15]. In oral SCC, perlecan biosynthesis switches parenchymal cells to stromal cells with invasion capacities; thus, perlecan deposition is considered a critical histological hallmark of invasion in oral SCC, which lacks the muscularis mucosae seen from the esophagus to the rectum [15]. However, the significance of ECM-rich stroma produced by tumor cells themselves in the function of salivary pleomorphic adenoma cells remains unknown.

To evaluate the cellular state under hypoxic conditions, the determination of the expression kinetics of hypoxia-inducible factor-1 (HIF-1), an oxygen-sensitive transcriptional activator, is important. HIF-1 consists of the constitutively expressed subunit, HIF-1β, and the oxygen-regulated subunit, HIF-1α. Under normal conditions, HIF-1α is degraded by the ubiquitin–proteasome pathway. Under hypoxic conditions, the HIF-1α subunit is stable and interacts with coactivators such as cAMP response element-binding protein/p300 to regulate target gene expression [16,17,18]. Thus, under hypoxic conditions, the degradation system of HIF is inhibited, and it functions as a transcription factor in the nucleus. Several studies have reported the role of HIF-1α in salivary gland tumors. HIF-1α activates Nidogen 1, a basement membrane component involved in the growth and metastasis of salivary adenoid cystic carcinoma (SACC), which promotes SACC cell migration via activation of the PI3K/AKT pathway and epithelial–mesenchymal transition [19]. Moreover, elevated levels of HIF-1α have been reported in epithelial-like cells of mucoepidermoid carcinoma, suggesting a link between the NOTCH1 signaling pathway and hypoxia [20].

In this study, we investigated the hypothesis that the stroma of salivary gland pleomorphic adenomas is hypoxic, enabling tumor cell proliferation without blood supply due to hypovascularization and ECM enrichment. Therefore, we investigated the relationship between hypoxia, represented by HIF-1α, and ECM molecules such as perlecan and FN, in pleomorphic adenoma tissues using our established SM-AP cell model to evaluate the mechanisms underlying pleomorphic adenoma cell survival in an ECM-rich, hypoxic environment.

## 2. Materials and Methods

### 2.1. Cell Lines

We used two previously established cell lines, SM-AP1 and SM-AP4, for the study [9]. SM-AP1 cells are polygonal in shape and immunohistochemically positive for keratin only, indicating duct epithelial or squamous cell differentiation, whereas SM-AP4 cells are spindle-shaped and positive for both keratin and S-100 proteins, indicating myoepithelial cell differentiation [9]. The cells were cultured in Dulbecco’s modified eagle medium (DMEM) (Thermo Fisher Scientific, Waltham, MA, USA) containing 10% fetal calf serum (FCS, Thermo Fisher Scientific), 50 µg/mL streptomycin, and 50 IU/mL penicillin (Thermo Fisher Scientific) and incubated at 37 °C in a humidified 5% carbon dioxide/95% air atmosphere for normoxia or nitrogen-based 1% oxygen for hypoxia, for 2 days (48 h).

### 2.2. Antibodies

Rabbit monoclonal antibodies against HIF-1α (Abcam, ab51608, Cambridge, UK, 1:500) and polyclonal antibodies against the mouse basement membrane-type perlecan core protein [21] and FN [12], raised in rabbits (diluted at 50 µg/mL), were used for immunoperoxidase staining, immunofluorescence, and Western blotting.

### 2.3. Immunohistochemistry

Immunohistochemical staining was performed using the ChemMate Envision^TM^ system (Dako, Glostrup, Denmark) as described elsewhere [9]. Reaction products were developed in 0.02% 3,3’-diaminobenzimine in 0.05 M Tris-HCl buffer (pH 7.6) containing 0.005% hydrogen peroxide. The sections were counterstained with hematoxylin. For control studies, primary antibodies were replaced by pre-immune rabbit IgGs.

### 2.4. Quantitative Reverse Transcription Polymerase Chain Reaction (qRT-PCR)

Total cellular RNAs were extracted from each cell type at 6 d after plating using TRIzol™ Reagent (Thermo Fisher Scientific). cDNA was synthesized from 5 µg of each RNA sample using the SuperScript Preamplification System^TM^ (Invitrogen, Thermo Fisher Scientific, Waltham, MA, USA) according to the manufacturer’s instructions. Quantitative RT-PCR was performed using SsoFast™ EvaGreen^®^ Supermix (Bio-Rad Laboratories, Inc., Hercules, CA, USA) and specific primers for HIF-1α, perlecan, and FN with a MiniOpticon Real-Time PCR Detection System CFB-3120 (Bio-Rad Laboratories). The expression level of β-actin was used to normalize variance. The PCR primer pairs used for each gene were as follows: HIF-1α, 5’-CCAGC AGACT CAAAT ACAAG AACC-3’, and 5’-TGTAT GTGGG TAGGA GATGG AGAT-3’; perlecan, 5’-CATGG GCTGA GGGCA TACG-3’, and 5’-TGTGC CCAGG CGTCG GAAC-3’; FN, 5’-GCCTG GTACA GAATA TGTAG TG-3’, and 5’-ATCCC AGCTG ATCAG TAGGC TGGTG-3’; β-actin, 5’-TCACC CACAC TGTGC CCATC TACGA-3’, and 5’-CAGCG GAACC GCTCA TTGCC AATGG-3’.

### 2.5. Immunofluorescence

SM-AP1 and SM-AP4 cells were plated at a concentration of 0.6 × 10^5^ onto chamber slides (Lab-Tek II, 2-well type, Nalge Nunc International, Naperville, IL, USA), and were fixed with 4% paraformaldehyde in PBS for 30 min and permeabilized with 0.2% Triton X-100 in phosphate-buffered saline (PBS) at room temperature for 20 min. To block non-specific binding, the cells were incubated with 5% milk protein in PBS and allowed to react with primary antibodies overnight at 4 °C [9]. The primary antibodies used were rabbit polyclonal antibodies against human HIF-1α (1:100), perlecan (1:100), and FN (1:50). The secondary antibodies used were rhodamine-conjugated goat anti-rabbit or mouse IgG (ICN Pharmaceuticals, Aurora, OH, USA, 1:50). For control studies, the primary antibodies were replaced with pre-immune rabbit IgGs. For perlecan and FN, at least a total of 15 cells were randomly selected after staining, and the average fluorescence intensity per unit area was measured using ImageJ 1.54f (National Institutes of Health, Bethesda, MD, USA).

### 2.6. Western Blotting

For the detection of HIF-1α, SM-AP1 and SM-AP4 cells, cultivated for up to 5 days under normoxia and/or hypoxic conditions for the last 2 days (48 h) starting from the 3rd day under hypoxic conditions were washed with cold PBS; their cell lysates were collected and divided into nuclear and cytoplasmic fractions using NE-PER nuclear and cytoplasmic extraction reagents (Thermo Fisher Scientific). A total of 2 μg proteins obtained from the fractions from SM-AP1 and 7 μg proteins from SM-AP4 were subjected to sodium dodecyl sulfate–polyacrylamide gel electrophoresis (SDS-PAGE) on 15% polyacrylamide slab gels and transferred to polyvinylidene difluoride (PVDF) membranes. Immunoreactants were detected using an ECL Plus^TM^ Western blotting analysis system (GE Healthcare UK Ltd., Buckinghamshire, UK).

### 2.7. RNA Interference (RNAi)

RNAi experiments were performed using the Stealth RNAi^TM^ small interfering RNA (siRNA) Duplex Oligoribonucleotides System (Invitrogen). We selected two different siRNAs from the three sequences targeting perlecan, FN, and HIF-1α. The siRNA sequences targeting perlecan were (#2) 5’-UGAAA GGACA ACCAC UUGGA UCCGG-3’; and (#3) 5’-UGAAG AGGAG GCCUC GAUGU AGAUG-3’. SM-AP1 and SM-AP4 cells transfected with perlecan-siRNA were designated as SM-AP1- or SM-AP4-perlecan-siRNA, respectively. The siRNA sequences targeting FN were: (#1) 5’-GCAGU GGCUG AAGAC ACAAG GAAAU-3’ and (#3) 5’-CAGUC AAAGC AAGCC CGGUU GUUAU-3’. SM-AP1 and SM-AP4 cells transfected with FN-siRNA were designated as SM-AP1- or SM-AP4-FN-siRNA, respectively. The siRNA sequences targeting HIF-1α were: (#2) 5’-GGGAU UAACU CAGUU UGAAC UAACU-3’ and (#3) 5’-GAAAU UCCUU UACAU AGCAA GACUU-3’. SM-AP1 cells transfected with HIF-1α-siRNA were designated as SM-AP1-HIF-1α-siRNA. Oligonucleotide concentration in the working solution was adjusted to 20 μM. Stealth RNAi^TM^ negative control low GC Duplex (sicont, Invitrogen), and sterile Milli-Q^TM^ water (cont) were used as negative controls. SM-AP cells were harvested in antibiotic- and FCS-free DMEM for 48 h and then transferred to 35 mm dishes (1 × 10^5^ cells/dish). Transfection of siRNA was conducted using Stealth^TM^ siRNAs and Lipofectamine^TM^ RNAiMAX (Invitrogen), according to the manufacturer’s instructions. The medium was renewed after 48 h to reduce cytotoxicity without reducing transfection efficiency. RNAi efficiencies were evaluated using RT-PCR, followed by cell proliferation and invasion analyses.

### 2.8. Cell Proliferation Assay

To investigate the effect of perlecan, FN, and HIF-1α on cell proliferation, SM-AP1, and SM-AP4 cells were seeded in 35 mm dishes at a concentration of 2.0 × 10^4^/well. After cultivation for 24 h, the cells were transfected with siRNA for 48 h and then collected using trypsin. The re-collected SM-AP cells were seeded again under normoxia at the same concentration, i.e., 2.0 × 10^4^ for perlecan-siRNA (#2, #3) and FN-siRNA (#1, #3) cells, and 6.0 × 10^4^ for HIF-1α-siRNA (#2, #3) cells, and the cell numbers were counted daily for 3 to 4 days. The same experiment was performed under hypoxia by seeding 6.0 × 10^4^ SM-AP1 cells. Cells with and without siRNA treatment (sicont and cont cells) were compared. The experiments were performed in triplicate.

### 2.9. Cell Migration Assay

SM-AP cells were seeded under normoxia at a cell concentration of 3.0 × 10^5^ in 1 mL DMEM (for perlecan), 2.5 × 10^5^ in 1 mL DMEM (for FN), and 0.6 × 10^5^ in 1 mL DMEM (for HIF-1α), each in Falcon^TM^ cell culture inserts bearing an 8 µm pore size polyethylene terephthalate membrane (BD Biosciences, San Jose, CA, USA). The plates were then incubated at 37 °C for 24 h. After incubation, the insert chamber was carefully removed with cotton swabs, fixed with 100% methanol, and stained with 1% toluidine blue; the bottom membrane filters were removed from the inserts and then mounted with cover glasses on glass slides with their bottom side up. Cells that migrated through the membrane filter were counted in three-unit fields of 4 mm^2^ and the mean values were calculated from triplicate experiments. To examine the effect of perlecan, FN, and HIF-1α on cell migration, cells with and without siRNA treatment were compared. The same experiment was performed under hypoxia by seeding 1.0 × 10^5^ SM-AP1 cells.

### 2.10. Xenografts in Nude Mice

SM-AP cells were transplanted into nude mice to measure the in vivo O_2_ concentrations in tumor masses using the oxygen electrode method with an oxygen monitor (BRC, Bioresearch Center, Tokyo, Japan). Cells at a concentration of 1.0 × 10^6^ in 0.2 mL culture media were injected into the lateral back of female BALB/c (nu/nu) mice at 3 to 5 weeks of age. The animals were housed in clean boxes in a hygienic and ventilated animal room and maintained under constant conditions (at 22 °C and in a 12 h light/dark cycle) with free access to sterilized solid food and autoclaved water. When tumors reached sizes of approximately 8–17 mm in diameter, they were surgically removed from the animals under anesthesia. The excised tumor tissues were fixed in 10% formalin for 24 h at 4 °C and embedded in paraffin. Serial sections, cut at 5 µm thickness, from the paraffin blocks were stained with hematoxylin and eosin and with immunoperoxidase for HIF-1α, perlecan, and FN, and then examined histologically.

### 2.11. Statistical Analysis

Cell count data from the cell proliferation and migration assays were analyzed using the Student’s *t*-test. For the analysis of perlecan and FN fluorescence intensity, the t-test or Mann–Whitney test was performed after assessing normality using GraphPad Prism 9 software (GraphPad Software, Inc., La Jolla, CA, USA). A *p*-value of less than 0.01 and/or 0.05 was considered statistically significant.

## 3. Results

### 3.1. Hypoxia-Induced HIF-1α in SM-AP Cells

**Figure 1:**
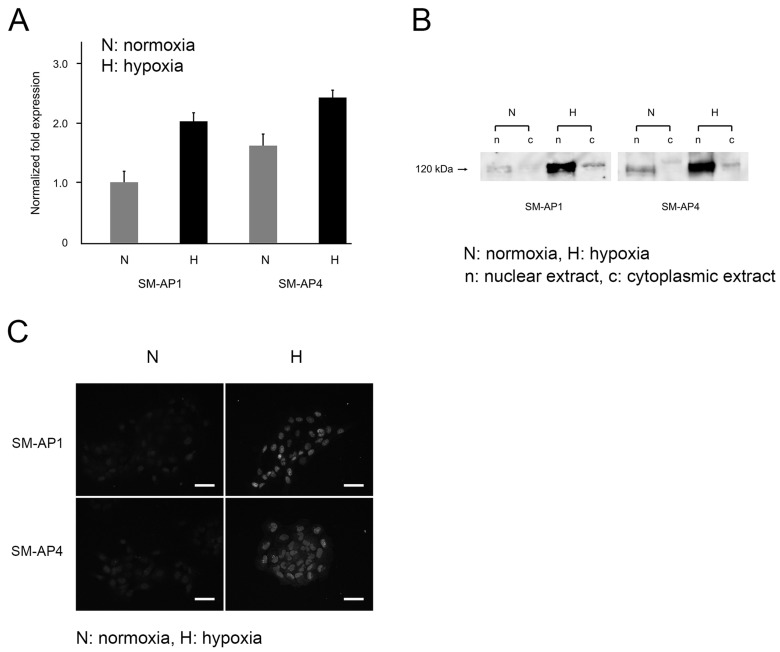
Comparative expression levels of HIF-1α in SM-AP1 and SM-AP4 cells under normoxia and hypoxia at 48 h of culture determined by real-time quantitative reverse transcription polymerase chain reaction (qRT-PCR) (**A**), Western blotting (**B**) and immunofluorescence (**C**), Scale bars, 100 µm. SM-AP1 and SM-AP4 cells showed higher HIF-1α mRNA (**A**) and protein (**B**) levels under hypoxia than under normoxia. Western blotting and immunofluorescence showed signals for HIF-1α were found in the nucleus of SM-AP1 and SM-AP4 cells under hypoxia, whereas a slight nuclear HIF-1α signal was detected in the cells under normoxia (**B**,**C**).

Hypoxia increased HIF-1α mRNA levels by 2-fold in SM-AP1 cells and by 1.5-fold in SM-AP4 cells compared to normoxia (Figure 1A). Protein levels of HIF-1α were also enhanced in SM-AP1 and SM-AP4 cells, especially in the nuclear fraction, under hypoxic conditions than in normoxic conditions (Figure 1B, Supplementary Figure S1). Immunofluorescence signals for HIF-1α were clearly localized in the nucleus of SM-AP1 and SM-AP4 cells under hypoxia (Figure 1C), whereas a slight nuclear HIF-1α signal was detected under normoxia (Figure 1C). The kinetics of HIF-1α expression indicated that SM-AP cells were responsive to hypoxic stress.

### 3.2. Increased Expression of Perlecan and FN in SM-AP Cells under Hypoxic Conditions

**Figure 2:**
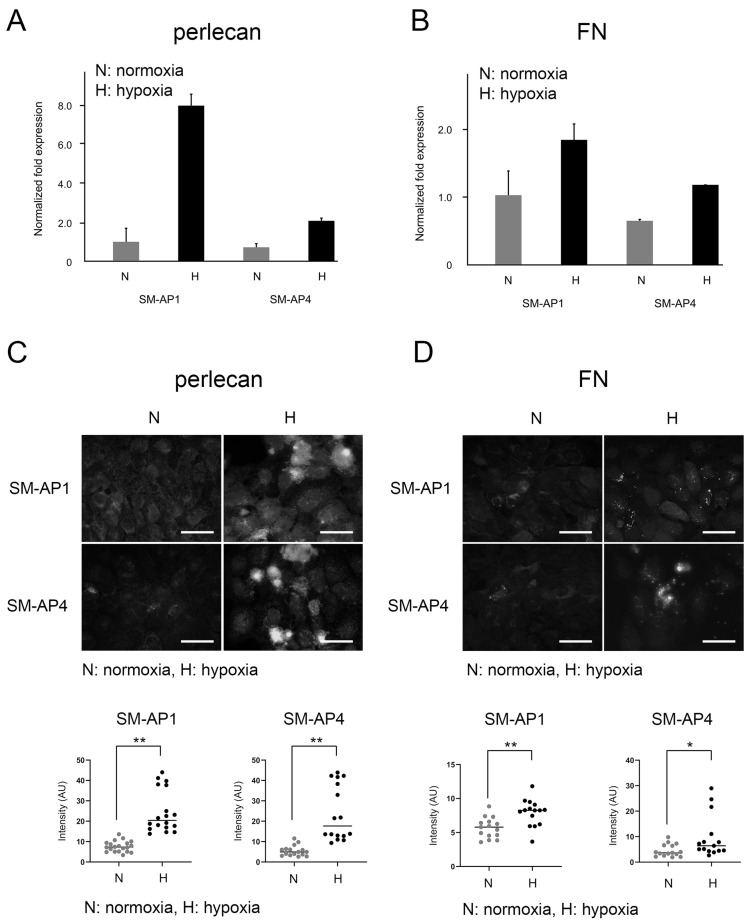
Comparative expression levels of perlecan and FN under normoxia and hypoxia at 48 h of culture, determined by qRT-PCR ((**A**), perlecan, (**B**), FN), and immunofluorescence ((**C**), perlecan, (**D**), FN), Scale bars, 100 µm. * *p* < 0.05. ** *p* < 0.01. SM-AP1 and SM-AP4 cells showed higher perlecan (**A**) and FN (**B**) mRNA expression levels under hypoxia than under normoxia. Immunofluorescence signals for perlecan and FN in SM-AP1 and SM-AP4 cells were observed like dots, mainly in the periphery of cells under hypoxia, whereas weak perlecan and FN signals were observed in SM-AP1 and SM-AP4 cells under normoxia compared to that in hypoxia (**C**,**D**). Brightness of immunofluorescence from perlecan and FN was predominantly higher under hypoxia than under normoxia (**C**,**D**).

The mRNA expression levels of perlecan and FN were increased by approximately 8-fold and 1.8-fold, respectively, in SM-AP1 cells, and by approximately 3-fold and 1.8-fold in SM-AP4 cells under hypoxia (Figure 2A,B). Immunofluorescence signal for perlecan was strongly observed like dots mainly in the periphery of SM-AP1 and SM-AP4 cells under hypoxia, suggesting their intracellular localization, although a weaker signal was observed under normoxia than under hypoxia (Figure 2C). Diffuse FN signal was observed like dots mainly in the cell periphery and perinuclear area of cells under hypoxia, suggesting their cell surface and/or intracellular localization, whereas very little FN signal was observed, mainly in the peri-nuclear area, under normoxia (Figure 2D). The brightness of immunofluorescence from perlecan and FN was predominantly greater under hypoxia than under normoxia (Figure 2C,D).

### 3.3. Perlecan Promoted Proliferation and Inhibited Invasion of SM-AP Cells

**Figure 3:**
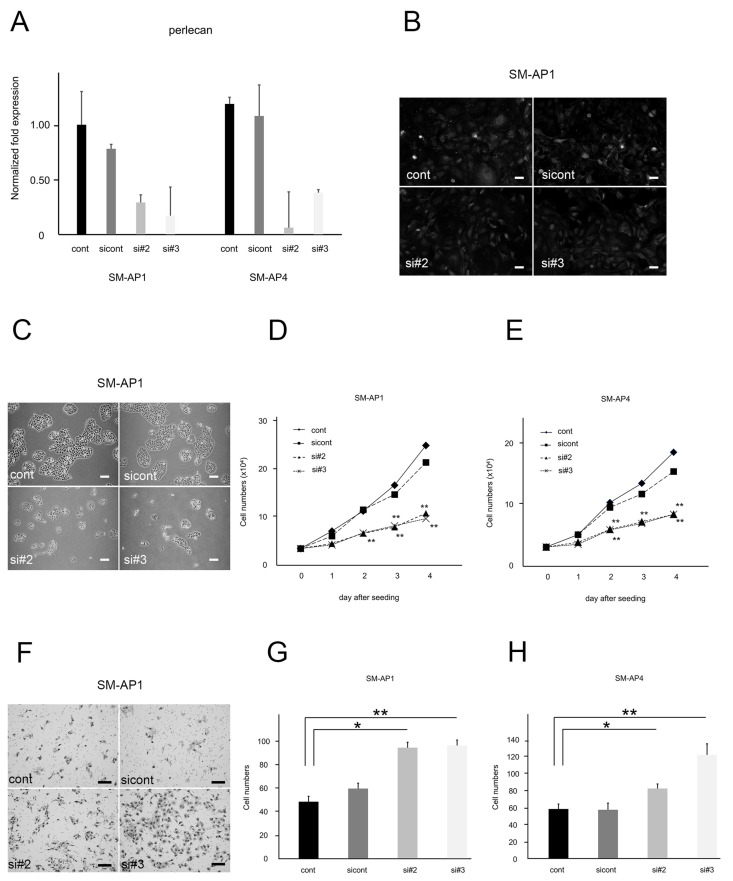
Comparison of proliferation and invasion between SM-AP1 and SM-AP4 control cells and SM-AP1- and SM-AP4-perlecan-siRNA cells. (**A**) Perlecan mRNA levels in SM-AP1 and SM-AP4-control cells (cont), SM-AP1 and SM-AP4-perlecan-sicontrol cells (sicont), and SM-AP1- and SM-AP4-perlecan-siRNA (si#2, si#3) cells. (**B**) Perlecan immunofluorescence in SM-AP1 cells. (**C**–**E**) Comparative proliferation modes between the cells in the presence and absence of perlecan. (**C**) Proliferating SM-AP1 on four days after seeding. (**D**) Comparative growth curves across SM-AP1-perlecan-cont, SM-AP1-perlecan-sicont and SM-AP1-perlecan-siRNA (#2, #3) cells. Effect of perlecan-siRNA, SM-AP1-perlecan-cont (diamond), SM-AP1-perlecan-sicont (square), SM-AP1-perlecan-si#2 (triangle), and SM-AP1-perlecan-si#3 (cross). (**E**) Comparative growth curves across SM-AP4-perlecan-cont, SM-AP4-perlecan-sicont and SM-AP4-perlecan-siRNA (#2, #3) cells. Effect of perlecan-siRNA, SM-AP4-perlecan-cont (diamond), SM-AP4-perlecan-sicont (square), SM-AP4-perlecan-si#2 (triangle), and SM-AP4-perlecan-si#3 (cross). (**F**–**H**) Comparative migration modes between the cells in the presence and absence of perlecan. (**F**) Migrating cells through membrane filters after 24 h, stained with toluidine blue. (**G**) Counts of migrating cells as shown in panel f at 24 h, SM-AP1-perlecan-cont (black-washed box), SM-AP1-perlecan-sicont (dark gray painted box), SM-AP1-perlecan-si#2 (light gray painted box), and SM-AP1-perlecan-si#3 (white-washed box). (**H**) Counts of migrating cells as shown at 24 h, SM-AP4-perlecan-cont (black-washed box), SM-AP4-perlecan-sicont (dark gray painted box), SM-AP4-perlecan-si#2 (light gray painted box), and SM-AP4-perlecan-si#3 (white-washed box). (**B**,**C**,**F**), Scale bars, 100 µm. * *p* < 0.05. ** *p* < 0.01. Perlecan mRNA level was suppressed in SM-AP1- and SM-AP4-perlecan-siRNA (si#2, si#3) cells, but remained unchanged in sicont, when compared to β-actin, an internal control (**A**). Immunofluorescence signal for perlecan was reduced by treatment with perlecan-siRNA (#2, #3) (**B**). SM-AP1 and SM-AP4 cell proliferation was suppressed by treatment with perlecan-siRNA (**C**–**E**). SM-AP1 and SM-AP4 cells grew more than twice as much as SM-AP1- and SM-AP4-perlecan-siRNA (#2, #3) cells on days 2 to 4 after seeding (**C**–**E**). SM-AP1- and SM-AP4-perlecan-siRNA (#2, #3) cells migrated faster than SM-AP1 and SM-AP4 sicont cells, and their migration was accelerated by the absence of perlecan (**F**–**G**).

Since ECM expression was increased under hypoxic conditions, we investigated the effect of siRNA-mediated suppression of ECM expression on SM-AP cell function. The two siRNA sequences (#2 and #3) for perlecan were confirmed by qRT-PCR to be effective in suppressing perlecan gene expression. Perlecan mRNA expression levels were lower in SM-AP1- and SM-AP4-perlecan-siRNA cells than in Stealth RNAi negative control cells (sicont) and control cells (cont) on day 3 after transfection (Figure 3A). Immunofluorescence revealed that perlecan protein levels were also reduced in SM-AP1-perlecan-siRNA cells than in control cells (Figure 3B). We compared cell numbers across SM-AP1- and SM-AP4-perlecan-siRNA, sicont, and cont for 4 days after plating, and observed SM-AP1 and SM-AP4 cell growth to be significantly inhibited by perlecan siRNA (*p* < 0.01, Figure 3C–E). The results suggested that perlecan signaling plays the role of growth enhancer, hence contributing to the proliferation of SM-AP1 and SM-AP4 cells. In contrast, the migration of SM-AP1 and SM-AP4 cells increased significantly 24 h after return seeding when treated with perlecan-si-RNA for the migration assay (*p* < 0.05, *p* < 0.01, Figure 3F–H). These results indicated that perlecan plays contradictory roles in proliferation and invasion in both SM-AP1 and SM-AP4 cells.

### 3.4. FN Promoted Proliferation of SM-AP Cells but Exerted Differential Effects on Invasion in SM-AP1 and SM-AP4 Cells

**Figure 4:**
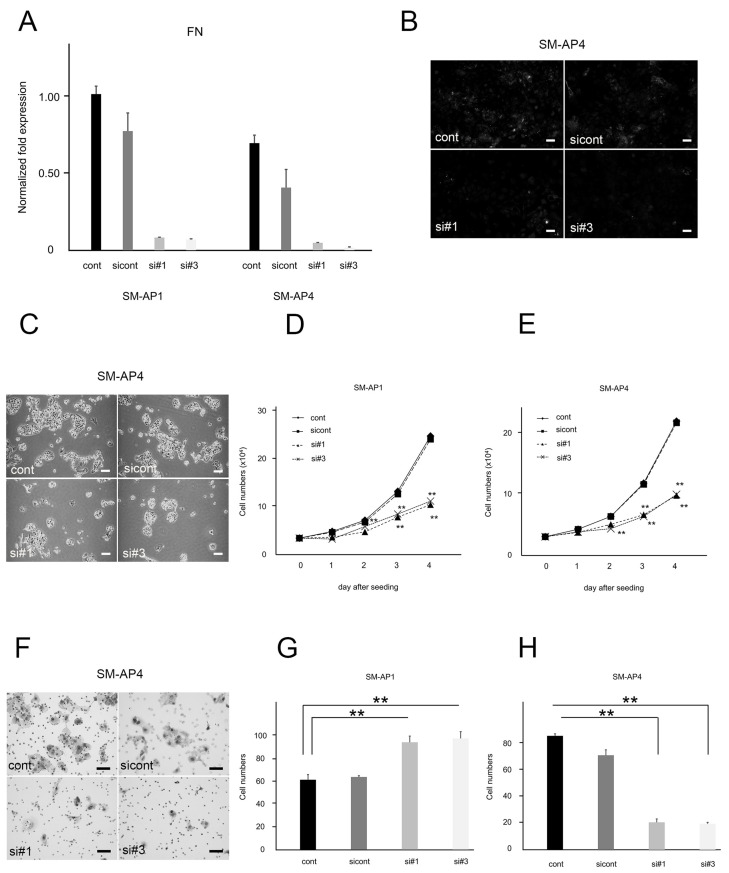
Comparison of proliferation and invasion between SM-AP1 and SM-AP4 control and SM-AP1- and SM-AP4-FN-siRNA cells. (**A**) FN mRNA levels in SM-AP1- and SM-AP4-cont, SM-AP1- and SM-AP4-FN-sicont, and SM-AP1- and SM-AP4-FN-siRNA (si#1, si#3) cells. (**B**) Immunofluorescence for FN in SM-AP4 cells. (**C**–**E**) Comparative proliferation modes between SM-AP1 and SM-AP4 cells in the presence and absence of FN. (**C**) SM-AP4 cell proliferation at four days after seeding. (**D**) Comparative growth curves across SM-AP1-FN-cont, SM-AP1-FN-sicont, and SM-AP1-FN-siRNA (#1, #3) cells. Effect of FN-siRNA, SM-AP1-FN-cont (diamond), SM-AP1-FN-sicontrol (square), SM-AP1-FN-si#1 (triangle), and SM-AP1-FN-si#3 (cross). (**E**) Comparative growth curves across SM-AP4-FN-cont, SM-AP4-FN-sicont, and SM-AP4-FN-siRNA (#1, #3) cells. Effect of FN-siRNA, SM-AP4-FN-cont (diamond), SM-AP4-FN-sicont (square), SM-AP4-FN-si#1 (triangle), and SM-AP4-FN-si#3 (cross). (**F**–**H**) Comparative migration modes between SM-AP1 and SM-AP4 cells in the presence and absence of FN. (**F**) Migrating cells through membrane filters after 24 h staining with toluidine blue. (**G**) Counts of migrating cells as shown in panel f at 24 h, SM-AP1-FN-cont (black-washed box), SM-AP1-FN-sicont (dark gray painted box), SM-AP1-FN-si#1 (light gray painted box), and SM-AP1-FN-si#3 (white-washed box). (**H**) Counts of migrating cells as shown at 24 h, SM-AP4-FN-cont (black-washed box), SM-AP4-FN-sicont (dark gray painted box), SM-AP4-FN-si#1 (light gray painted box), and SM-AP4-FN-si#3 (white-washed box). (**B**,**C**,**F**), Scale bars, 100 µm. ** *p* < 0.01. FN mRNA level was suppressed in SM-AP1- and SM-AP4-FN-siRNA (#1, #3) cells, but remained unchanged in sicont, when compared with β-actin, an internal control (**A**). Immunofluorescence signal for FN was reduced by treatment with FN-siRNA (#1 and #3) (**B**). SM-AP1 and SM-AP4 cell proliferation was suppressed by treatment with FN-siRNA (**C**–**E**). SM-AP1 and SM-AP4 cells grew more than twice as much as SM-AP1- and SM-AP4-FN-siRNA (#1, #3) cells on days 2 to 4 after seeding (**C**–**E**). SM-AP1-FN-siRNA (#1, #3) cells migrated faster than SM-AP1 cells and cont (**G**), but SM-AP4-FN-siRNA (#1, #3) cells migrated slower than SM-AP4 cells and cont (**F**,**H**).

The two siRNA sequences (#1 and #3) for FN were confirmed to be similarly effective in suppressing FN gene expression using qRT-PCR. FN mRNA levels were lower in SM-AP1- and SM-AP4-FN-siRNA cells than in Stealth RNAi negative control cells on day 3 after transfection (Figure 4A). The immunofluorescence signal for FN was also reduced in SM-AP4-FN-siRNA cells than in control cells (Figure 4B). Cell number comparison across SM-AP1- and SM-AP4-FN-siRNA, sicont, and cont for 4 days after plating revealed that SM-AP1 and SM-AP4 cell growth was significantly inhibited by siRNA against FN (*p* < 0.01, Figure 4C–E). The results suggested that FN signaling enhanced the proliferation of SM-AP1 and SM-AP4 cells. Migration assays showed significantly increased SM-AP1 migrating cells (*p* < 0.01, Figure 4G), but significantly reduced SM-AP4 migrating cells (*p* < 0.01, Figure 4F,H) 24 h after return seeding upon treatment with FN-siRNA. In contrast to perlecan, FN played different roles in migration depending on the cell type (SM-AP1 vs. SM-AP4 cells).

### 3.5. SM-AP Cells Promoted ECM Synthesis in Xenografts under Hypoxia

**Figure 5:**
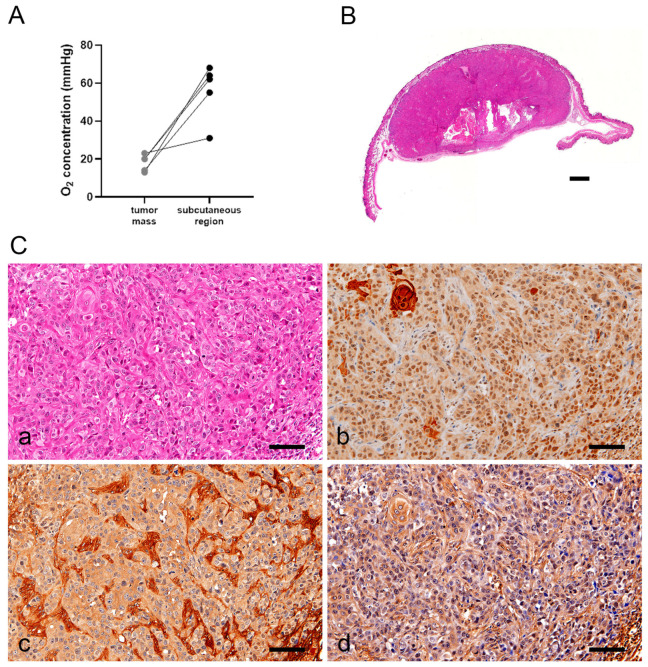
Transplanted tumors of SM-AP1 cells in nude mice. Comparison of oxygen levels in the transplanted tumor mass that in the surrounding subcutaneous tissue in a nude mouse (**A**); cut surface view of a subcutaneous SM-AP1 tumor (**B**); histopathology of transplanted SM-AP1 tumors (**C**). H&E staining (**C**(**a**)); immunoperoxidase staining for HIF-1α (**C**(**b**)), perlecan (**C**(**c**)) and FN (**C**(**d**)), hematoxylin counterstain. (**B**), Scale bar, 1000 µm; (**C**(**a**–**d**)), Scale bars, 100 µm. Transplanted tumor masses of SM-AP1 cells show lower O_2_ concentration than that of subcutaneous region (**A**) SM-AP1 cells formed subcutaneous tumors measuring approximately 15 mm in diameter in nude mice within one to four months (**B**). Histopathological analysis revealed that the tumors were squamous cell carcinomas with definite tendencies towards keratinization, and the stroma was wide, hyaline, and poorly vascularized (**C**(**a**)). The nuclei of the tumor cells were positive for HIF-1α (**C**(**b**)). The hyaline stroma was immunopositive for perlecan (**C**(**c**)) and FN (**C**(**d**)).

The oxygen concentration of the five transplanted tumor masses created by implanting SM-AP1 cells into female BALB/c (nu/nu) nude mice was lower than that in the subcutaneous region (*p* < 0.05, Figure 5A). SM-AP1 cells formed subcutaneous tumors measuring approximately 15 mm in diameter in nude mice within one to four months (Figure 5B). Histopathological analysis revealed that the tumors were squamous cell carcinomas with definite tendencies towards keratinization and that the stroma was wide, hyaline, and with poor vascularity (Figure 5C(a)). The nuclei of the tumor cells within tumor foci were uniformly positive for HIF-1α (Figure 5C(b)). Perlecan was immunopositive in the hyaline stroma and cytoplasm (Figure 5C(c)). FN was present in the cytoplasm and intercellular space (Figure 5C(d)). Thus, hypoxia was maintained in the transplanted tumors, confirming the nuclear localization of HIF-1α, and promotion of extracellular matrix synthesis by SM-AP1 cells.

### 3.6. Inhibition of HIF-1α Suppressed the Proliferation, Migration, and ECM Synthesis in SM-AP1 Cells

**Figure 6:**
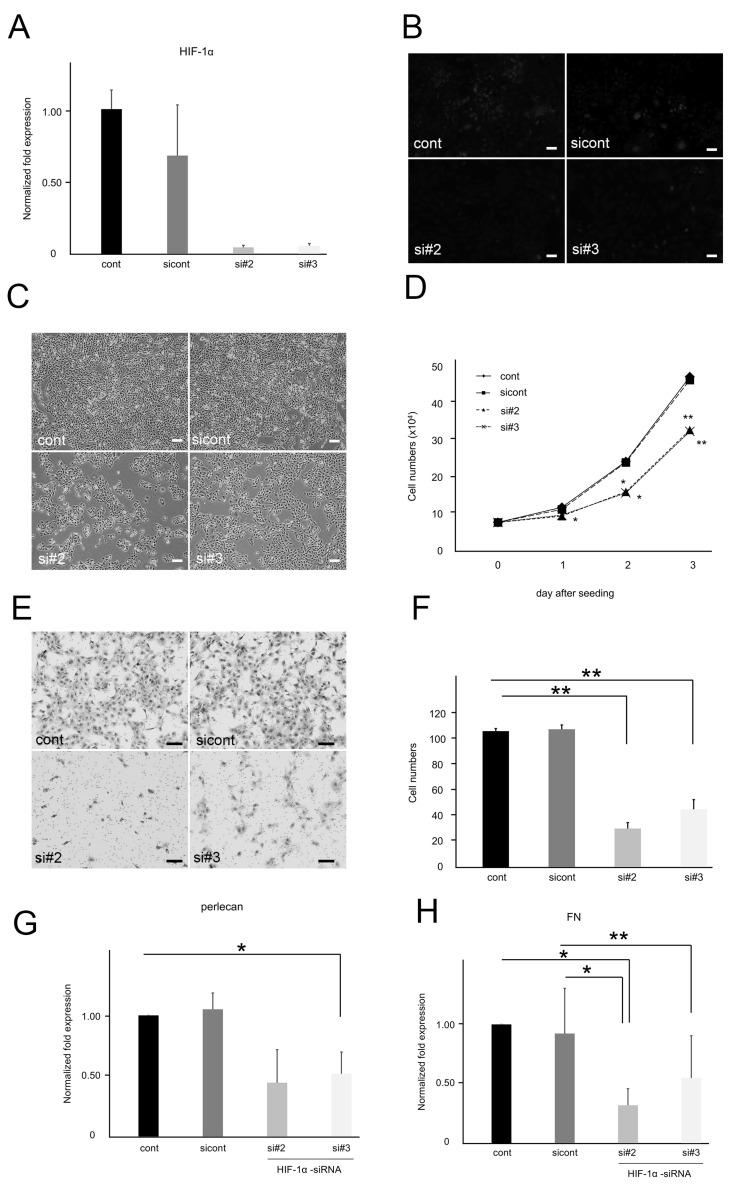
Comparison of proliferation and invasion between SM-AP1 and SM-AP1-HIF-1α-siRNA cells. (**A**) HIF-1α mRNA levels in SM-AP1-cont, SM-AP1-HIF-1α-sicont, and SM-AP1-HIF-1α-siRNA (si#2, si#3) cells. (**B**) Immunofluorescence for HIF-1α in SM-AP1 cells. (**C**,**D**) Comparative proliferation modes between SM-AP1 cells in the presence and absence of HIF-1α. (**C**) SM-AP1 cell proliferation four days after seeding. (**D**) Comparative growth curves between SM-AP1-HIF-1α-cont, SM-AP1-HIF-1α-sicont, and SM-AP1-HIF-1α-siRNA (#2, #3) cells. Effect of HIF-1α-siRNA, SM-AP1-HIF-1α-cont (diamond), SM-AP1-HIF-1α-sicont (square), SM-AP1-HIF-1α-si#2 (triangle), and SM-AP1-HIF-1α-si#3 (cross). (**E**,**F**) Comparative migration modes between SM-AP1 cells in the presence and absence of HIF-1α. (**E**) Migrating SM-AP1 cells through membrane filters after 24 h staining with toluidine blue. (**F**) Counts of migrating cells as shown in panel F at 24 h, SM-AP1 (black-washed box), SM-AP1-HIF-1α-sicont (dark gray painted box), SM-AP1-HIF-1α-si#2 (light gray painted box), and SM-AP1-HIF-1α-si#3 (white-washed box). (**G**) Perlecan mRNA levels in SM-AP1 and SM-AP1-HIF-1α-siRNA (#2, #3), SM-AP1-HIF-1α-cont (black-washed box), SM-AP1-HIF-1α-sicont (dark gray painted box), SM-AP1-HIF-1α-si#2 (light gray painted box), and SM-AP1-HIF-1α-si#3 (white-washed box) cells. (**H**) FN mRNA levels in SM-AP1 and SM-AP1-HIF-1α-siRNA (#2, #3) cells, SM-AP1-HIF-1α-cont (black-washed box), SM-AP1-HIF-1α-sicont (dark gray painted box), SM-AP1-HIF-1α-si#2 (light gray painted box), and SM-AP1-HIF-1α-si#3 (white-washed box). (**B**), (**C**,**E**), Scale bars, 100 µm. * *p* < 0.05. ** *p* < 0.01. HIF-1α mRNA level was suppressed in SM-AP1-HIF-1α-siRNA (#2, #3) cells, but remained unchanged in sicont, when compared to β-actin, an internal control (**A**). Immunofluorescence signal for HIF-1α was reduced by treatment with HIF-1α-siRNA (**B**). SM-AP1 cell proliferation was suppressed by treatment with HIF-1α-siRNA (**C**,**D**). SM-AP1 cells grew twice as much as SM-AP1-HIF-1α-siRNA (#2, #3) cells on days 1 to 3 after seeding (**D**). SM-AP1-HIF-1α-siRNA (#2, #3) cells migrated slower than SM-AP1 cells and cont, and their migration was suppressed by the absence of HIF-1α (**E**,**F**). There were obvious suppressions in perlecan (**G**) and FN (**H**) mRNA expression levels in SM-AP1-HIF-1α-siRNA (#2-#3) cells, while no change was observed in control cells.

We found that SM-AP1 promoted ECM synthesis under hypoxia, whereas the inhibition of ECM synthesis suppressed cell proliferation and promoted cell migration. Conversely, we examined the effects of HIF-1α inhibition on proliferation, migration, and ECM synthesis in SM-AP1 cells. The two siRNA sequences (#2 and #3) for HIF-1α were similarly effective in suppressing HIF-1α gene expression by qRT-PCR. HIF-1α mRNA levels were decreased in SM-AP1-HIF-1α-siRNA cells than in Stealth RNAi negative cont and cont on day 3 after transfection (Figure 6A). Moreover, a little HIF-1α expression was detected under normoxia, and immunofluorescence revealed HIF-1α expression levels to not be detected in SM-AP1-HIF-1α-siRNA cells (Figure 6B). Cell number comparison across SM-AP1-HIF-1α-siRNA, and sicont and cont cells, 3 days after plating, revealed that SM-AP1 cell growth was significantly inhibited by HIF-1α siRNA (*p* < 0.05, *p* < 0.01, Figure 6C,D). These results suggested that HIF-1α signaling also plays a role in SM-AP1 cell proliferation and that SM-AP1 cell proliferation is promoted in the presence of HIF-1α. In migration assays, SM-AP1 invading cells were significantly reduced 24 h after return seeding upon treatment with HIF-1α-siRNA (*p* < 0.01, Figure 6E,F), suggesting that SM-AP1 cell migration was reduced upon suppression of HIF-1α expression. Synthesis of ECM components such as perlecan (*p* < 0.05, Figure 6G) and FN (*p* < 0.05, *p* < 0.01, Figure 6H) in SM-AP1 cells was also suppressed by HIF-1α-siRNA, suggesting that ECM synthesis under hypoxic conditions may be mediated by HIF-1α.

### 3.7. Inhibition of HIF-1α and ECM Synthesis under Hypoxia Suppressed the Proliferation in SM-AP1 Cells

**Figure 7:**
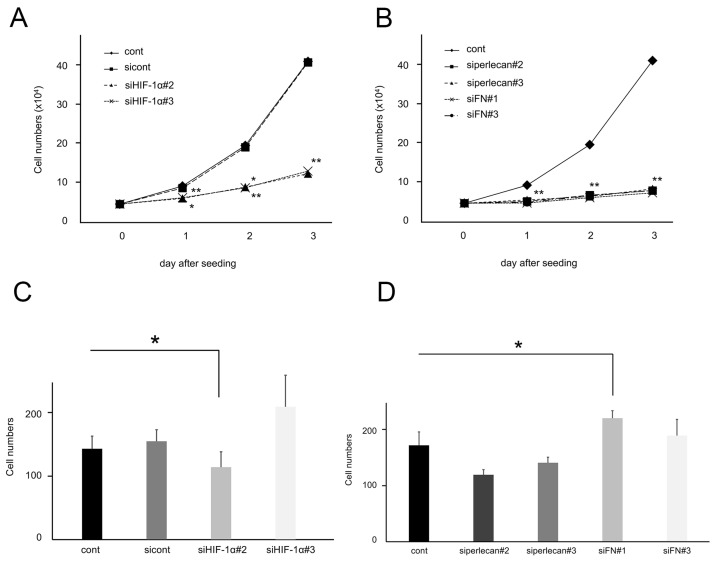
Comparison of proliferation and migration between SM-AP1 and SM-AP1-HIF-1α-siRNA or perlecan-siRNA or FN-siRNA cells under hypoxia. (**A**,**B**) Comparative proliferation modes between SM-AP1 cells in the presence and absence of HIF-1α or perlecan or FN under hypoxia. (**A**) Comparative growth curves between SM-AP1-cont, SM-AP1-sicont, SM-AP1-HIF-1α-siRNA (#2, #3) cells under hypoxia. Effect of HIF-1α-siRNA, SM-AP1-cont (diamond), SM-AP1-sicont (square), SM-AP1-HIF-1α-si#2 (triangle), and SM-AP1-HIF-1α-si#3 (cross). (**B**) Comparative growth curves between SM-AP1-cont, SM-AP1-sicont, SM-AP1-perlecan-siRNA (#2, #3) and SM-AP1-FN-siRNA (#1, #3) cells under hypoxia. Effect of perlecan or FN-siRNA, SM-AP1-cont (diamond), SM-AP1-perlecan-si#2 (square), SM-AP1-siperlecan-si#3 (triangle), SM-AP1-FN-si#1 (cross) and SM-AP1-FN-si#3 (circle). (**C**,**D**) Comparative migration modes between SM-AP1 cells in the presence and absence of HIF-1α or perlecan or FN under hypoxia. (**C**) Counts of migrating cells between SM-AP1-cont (black-washed box), SM-AP1-sicont (dark gray painted box), SM-AP1-HIF-1α-si#2 (light gray painted box), and SM-AP1-HIF-1α-si#3 (white-washed box). (**D**) Counts of migrating cells between SM-AP1 (black-washed box), SM-AP1-perlecan-si#2 (dark gray painted box), SM-AP1-perlecan-si#3 (gray painted box), SM-AP1-FN-si#1 (light gray painted box), and SM-AP1-FN-si#3 (white-washed box). * *p* < 0.05. ** *p* < 0.01. SM-AP1 cells proliferated 3-fold more than SM-AP1-HIF-1α-si#2 and SM-AP1-HIF-1α-si#3 cells (**A**) and 4-fold more than SM-AP1-perlecan-si#2, SM-AP1-perlecan-si#3, SM-AP1-FN-si#1, and SM-AP1-FN-si#3 cells (**B**) on day 3 days after seeding even under hypoxia. SM-AP1-HIF-1α-si#2 cells migrated slower (**C**) and SM-AP1-FN-si#1 cells migrated faster (**D**) than SM-AP1-cont cells under hypoxia.

We found that SM-AP1 cells also suppressed the proliferation by inhibition of HIF-1α and ECM synthesis under hypoxia. We examined the effects of HIF-1α and ECM synthesis inhibition on SM-AP1 cell proliferation and migration under hypoxia. In proliferation assays, SM-AP1 cell growth was also significantly inhibited by all HIF-1α siRNA (*p* < 0.05, *p* < 0.01, Figure 7A), perlecan siRNA, and FN siRNA (*p* < 0.01, Figure 7B) under hypoxia. In the migration assay, SM-AP1-invading cells were significantly reduced 24 h after reversion seeding when treated with HIF-1α-siRNA#2 under hypoxia (*p* < 0.05, Figure 7C) and significantly increased when treated with FN-siRNA#1 under hypoxia (*p* < 0.05, Figure 7D).

## 4. Discussion

In this study, we demonstrated for the first time that SM-AP cells, established from salivary gland pleomorphic adenoma, maintain high levels of HIF-1α under hypoxic conditions and show enhanced proliferation by activating their ECM biosynthesis. Based on our previous findings, we focused on perlecan and FN, the ECM molecules secreted by SM-AP cells, which are especially abundant in the stroma of pleomorphic adenoma. Although perlecan has been reported to play an important role in regulating tumor cell metastasis by mediating cell adhesion and regulating the activity of several growth and motility factors, the mechanisms underlying these effects remain to be elucidated [22,23,24]. Moreover, since the controversial claim that FN functions as an endothelial-binding ligand for tumor cells in the blood to mediate and promote colony formation and metastasis to the lung, accumulating evidence has revealed that FN expression in cancer critically contributes to tumor grade, metastasis, and poor patient prognosis [25]. We confirmed that the expression of perlecan and FN mRNA and protein was maintained at high levels under hypoxic conditions in SM-AP1 and SM-AP4 cells and that tumors created by transplanting SM-AP cells into nude mice also maintained hypoxia and promoted the synthesis of perlecan and FN. Similar to our results, Asplund A et al. reported that proteoglycans were secreted by macrophages in hypoxic regions of atherosclerotic lesions and that perlecan expression was regulated by HIF-1α [26]. In their chapter on hypoxia-induced re-expression of FN in tumor cells and cancer metastasis, Lin et al. summarized that HIF activation leads to endogenous FN synthesis [25]. In addition, similar to our results, another study reported that hypoxia stimulated fibronectin autocrine secretion in squamous cell carcinoma cells [27].

Our results suggested that SM-AP1 promotes ECM synthesis under hypoxia and that inhibition of ECM synthesis under normoxia would inhibit cell proliferation and promote cell migration. In contrast, suppression of HIF-1α under normoxia inhibited cell proliferation but reversed cell migration in SM-AP1 cells. Inhibition of HIF-1α also suppressed ECM synthesis, suggesting that ECM synthesis may be mediated by HIF-1α. The results of cell migration may reflect the effects of HIF-1α-mediated promotion of ECM synthesis under hypoxia and inhibition of ECM synthesis by siRNA. Savorè C et al. reported decreased cell proliferation rate and reduced tumor size due to an inhibited response to heparin-binding growth factor and reported that perlecan is an essential ECM component involved in the tumor growth response in a prostate cancer cell line with perlecan knockdown [28]. An examination of the role of the two major basement membrane proteoglycans, agrin and perlecan, in oral cancer revealed that agrin and perlecan are highly expressed in oral SCC cells, and their knockdown suppressed cell migration and adhesion and increased resistance to cisplatin, making them potential therapeutic targets [29]. In addition, Sanderson reported that heparan sulfate proteoglycans act as inhibitors of cell invasion and at other times as promoters of cell invasion, and their function is determined by their location, the heparin-binding molecules they bind to, the presence of modifying enzymes, and the precise structural properties of the proteoglycans [22]. The role of FN expression in tumor cells is complex. In previous studies, FN expression in tumor cells was primarily considered to be a tumor suppressor; however, later it switched to a metastasis-promoting factor [25]. Ryu et al. suggested that hypoxia promotes oral squamous cell carcinoma cell invasion, which is elicited by the induction of α5 integrin and FN, the major factors involved in HIF-1α-dependent tumor cell invasion [27]. When SM-AP1 cell growth suppression was compared between SM-AP1-HIF-1α-siRNA and SM-AP1-perlecan/FN-siRNA, the latter was found to be relatively more effective. These results suggest that the promotion of SM-AP cell proliferation under hypoxia could be due to further enhancement of perlecan and FN production by HIF-1α. As for cell migration, the results of inhibition of ECM synthesis and HIF-1α were inconsistent; inhibition of FN synthesis under normoxia either promoted or inhibited cell migration, suggesting the possible involvement of a mechanism of ECM synthesis different from that of HIF-1α. This could be an area for future studies. Although epithelial–mesenchymal transition in PAs has been reported to likely be involved in PA development, chondrocyte differentiation, and malignant transformation, it has been pointed out that there are many unknowns, including the relationship with HIF1, and we think that the involvement of regulatory mechanisms of epithelial–mesenchymal transition in cell migration is another topic for future research [30,31].

Amelio et al. reported that depletion of p53 mutants in non-small cell lung cancer (NSCLC) suppresses the HIF-induced upregulation of ECM components, such as collagen type VIIa1 and laminin-γ2, resulting in the reduced tumorigenic potential of NSCLC cells, which in concert with HIF-1 affects ECM synthesis and the tumor microenvironment to promote cancer progression [32,33]. We had previously shown that SM-AP1 and SM-AP4 cells also possess the *p53* gene mutations, and similar mechanisms as those described by Amelio et al. could be involved; however, this needs to be investigated further [9].

## 5. Conclusions

Taken together, our findings suggested that SM-AP cells may function as important drivers of cell proliferation under hypoxic conditions by maintaining high levels of the hypoxia-sensitive factor HIF-1α, mainly in the nucleus, thereby promoting the synthesis of the extracellular substrates, perlecan, and fibronectin. Thus, the regulation of the biosynthesis of ECM molecules in the tumor microenvironment, which is considered to be a hypoxic environment, may be a new anti-tumor growth target. However, just as tumor cells are resistant to anti-angiogenic therapies, their resistance to hypoxic environments must also be considered, and understanding the complex mechanisms involved in tumor growth and resistance to therapy would be important in order to develop effective treatments [34]. Furthermore, in addition to the fact that this study was an in vitro experimental system using the SM-AP cell system, Cardoso et al. also reported that in their examination of HIF-1α levels as a hypoxia marker using surgical material from benign and malignant salivary gland neoplasms, no significant differences in HIF-1α expression were found [35]. Therefore, in addition to our in vitro experimental system using SM-AP cells, we believe that future studies using surgical materials are also needed.

## Data Availability

Not applicable.

## Supplementary Materials

The following supporting information can be downloaded at: https://www.mdpi.com/article/10.3390/biomedicines11112981/s1, Figure S1: The original blot images of Figure 1B in the manuscript. Western blotting data for HIF-1α, (**A**) SM-AP1, and (**B**) SM-AP4. Left to right: marker, Normoxia (Nuclear extract), Normoxia (Cytoplasmic extract), Hypoxia (Nuclear extract), Hypoxia (Cytoplasmic extract). HIF-1α protein levels of (**A**) SM-AP1 and (**B**) SM-AP4 were measured by densitometry of each band/intensity ratio using Image J.

## References

[1] Bell D., Bullerdiek J., Gnepp D.R., Schwartz M.R., Stenman G., Triantafyllou A., El-Naggar Adel K., Chan John K.C., Grandis Jennifer R., Takata T., Slootweg Piter J. (2017). Benign tumor (pleomorphic adenoma). WHO Classification of Head and Neck Tumors.

[2] Bishop Justin A., Thompson Lester D.R., Wakely P.E., Paul E., Weneb I. (2021). Tumors of the Salivary Glands: AFIP Atlas of Tumor and Non-Tumor Pathology, 5th ed.

[3] Stennert E., Guntinas-Lichius O., Klussmann J.P., Arnold G. (2001). Histopathology of pleomorphic adenoma in the parotid gland: A prospective unselected series of 100 cases. Laryngoscope.

[4] LiVolsi V.A., Perzin K.H. (1977). Malignant mixed tumors arising in salivary glands. part I. Carcinomas arising in benign tumors: A clinicopathologic study. Cancer.

[5] Di Palma S., Skálová A., Vanìèek T., Simpson R.H., Stárek I., Leivo I. (2005). Non-invasive (intracapsular) carcinoma ex pleomorphic adenoma: Recognition of focal carcinoma by HER-2/neu and MIB1 immunohistochemistry. Histopathology.

[6] Antony J., Gopalan V., Smith R.A., Lam A.K. (2012). Carcinoma ex pleomorphic adenoma: A comprehensive review of clinical, pathological and molecular data. Head Neck Pathol..

[7] Brandwein M., Huvos A.G., Dardick I., Thomas M.J., Theise N.D. (1996). Noninvasive and minimally invasive carcinoma ex mixed tumor: A clinicopathologic and ploidy of 12 patients with major salivary tumors of low (or no?) malignant potential. Oral. Surg. Oral. Med. Oral. Pathol. Radiol. Endod..

[8] Takeda Y. (1999). An immunohistochemical study of bizarre neoplastic cells in pleomorphic adenoma: Its cytological nature and proliferative activity. Pathol. Int..

[9] Maruyama S., Cheng J., Shingaki S., Tamura T., Asakawa S., Minoshima S., Shimizu Y., Shimizu N., Saku T. (2009). Establishment and characterization of pleomorphic adenoma systems: An in-vitro demonstration of carcinomas arising secondarily from adenomas in the salivary gland. BMC Cancer.

[10] Maruyama S., Cheng J., Yamazaki M., Liu A., Saku T. (2009). Keratinocyte growth factor colocalized with perlecan at the site of capsular invasion and vascular involvement in salivary pleomorphic adenomas. J. Oral. Pathol. Med..

[11] Raitz R., Martins M.D., Araújo V.C. (2003). A study of the extracellular matrix in salivary gland tumors. J. Oral. Pathol. Med..

[12] Cheng J., Saku T., Okabe H., Furthmayr H. (1992). Basement membranes in adenoid cystic carcinoma. An immunohistochemical study. Cancer.

[13] Mishra M., Naik V.V., Kale A.D., Ankola A.V., Pilli G.S. (2011). Perlecan (basement membrane heparan sulfate proteoglycan) and its role in oral malignancies: An overview. Indian J. Dent. Res..

[14] Mishra M., Chandavarkar V., Naik V.V., Kale A.D. (2013). An immunohistochemical study of basement membrane heparan sulfate proteoglycan (perlecan) in oral epithelial dysplasia and squamous cell carcinoma. J. Oral. Maxillofac. Pathol..

[15] Maruyama S., Shimazu Y., Kudo T., Sato K., Yamazaki M., Abé T., Cheng J., Aoba T., Saku T. (2014). Three-dimensional visualization of perlecan-rich neoplastic stroma induced concurrently with the invasion of oral squamous cell carcinoma. J. Oral. Pathol. Med..

[16] Ke Q., Costa M. (2006). Hypoxia-inducible factor-1 (HIF-1). Mol. Pharmacol..

[17] Cho H., Ahn D.R., Park H., Yang E.G. (2007). Modulation of p300 binding by posttranslational modifications of the C-terminal activation domain of hypoxia-inducible factor-1alpha. FEBS Lett..

[18] Dyson H.J., Wright P.E. (2016). Role of Intrinsic Protein Disorder in the Function and Interactions of the Transcriptional Coactivators CREB-binding Protein (CBP) and p300. J. Biol. Chem..

[19] Han N., Li X., Wang Y., Li H., Zhang C., Zhao X., Zhang Z., Ruan M., Zhang C. (2022). HIF-1α induced NID1 expression promotes pulmonary metastases via the PI3K-AKT pathway in salivary gland adenoid cystic carcinoma. Oral. Oncol..

[20] Branco D.C., da Costa N.M.M., Abe C.T.S., Kataoka M.S.D.S., Pinheiro J.J.V., Alves Júnior S.M. (2019). HIF-1α, NOTCH1, ADAM12, and HB-EGF are overexpressed in mucoepidermoid carcinoma. Oral. Surg. Oral. Med. Oral. Pathol. Oral. Radiol..

[21] Saku T., Furthmayr H. (1989). Characterization of the major heparan sulfate proteoglycan secreted by bovine aortic endothelial cells in culture. Homology to the large molecular weight molecule of basement membranes. J. Biol. Chem..

[22] Sanderson R.D. (2001). Heparan sulfate proteoglycans in invasion and metastasis. Semin. Cell Dev. Biol..

[23] Elgundi Z., Papanicolaou M., Major G., Cox T.R., Melrose J., Whitelock J.M., Farrugia B.L. (2020). Cancer metastasis. The role of the extracellular matrix and the heparan sulfate proteoglycan perlecan. Front. Oncol..

[24] Cruz L.A., Tellman T.V., Farach-Carson M.C. (2020). Flipping the Molecular Switch: Influence of Perlecan and Its Modifiers in the Tumor Microenvironment. Adv. Exp. Med. Biol..

[25] Lin T.C., Yang C.H., Cheng L.H., Chang W.T., Lin Y.R., Cheng H.C. (2019). Fibronectin in cancer: Friend or foe. Cells.

[26] Asplund A., Stillemark-Billton P., Larsson E., Rydberg E.K., Moses J., Hultén L.M., Fagerberg B., Camejo G., Bondjers G. (2010). Hypoxic regulation of secreted proteoglycans in macrophages. Glycobiology.

[27] Ryu M.H., Park H.M., Chung J., Lee C.H., Park H.R. (2010). Hypoxia-inducible factor-1alpha mediates oral squamous cell carcinoma invasion via upregulation of alpha5 integrin and fibronectin. Biochem. Biophys. Res. Commun..

[28] Savorè C., Zhang C., Muir C., Liu R., Wyrwa J., Shu J., Zhau H.E., Chung L.W., Carson D.D., Farach-Carson M.C. (2005). Perlecan knockdown in metastatic prostate cancer cells reduces heparin-binding growth factor responses in vitro and tumor growth in vivo. Clin. Exp. Metastasis.

[29] Kawahara R., Granato D.C., Carnielli C.M., Cervigne N.K., Oliveria C.E., Rivera C., Yokoo S., Fonseca F.P., Lopes M., Santos-Silva A.R. (2014). Agrin and perlecan mediate tumorigenic processes in oral squamous cell carcinoma. PLoS ONE.

[30] Tam S.Y., Wu V.W.C., Law H.K.W. (2020). Hypoxia-Induced Epithelial-Mesenchymal Transition in Cancers: HIF-1α and Beyond. Front. Oncol..

[31] Matsumiya-Matsumoto Y., Morita Y., Uzawa N. (2022). Pleomorphic Adenoma of the Salivary Glands and Epithelial-Mesenchymal Transition. J. Clin. Med..

[32] Amelio I., Melino G. (2015). The p53 family and the hypoxia-inducible factors (HIFs): Determinants of cancer progression. Trends Biochem. Sci..

[33] Amelio I., Mancini M., Petrova V., Cairns R.A., Vikhreva P., Nicolai S., Marini A., Antonov A.A., Le Quesne J., Baena Acevedo J.D. (2018). p53 mutants cooperate with HIF-1 in transcriptional regulation of extracellular matrix components to promote tumor progression. Proc. Natl. Acad. Sci. USA.

[34] Ribatti D., Solimando A.G., Pezzella F. (2021). The anti-VEGF(R) drug discovery legacy: Improving attrition rates by breaking the vicious cycle of angiogenesis in cancer. Cancers.

[35] Cardoso C.M., de Jesus S.F., de Souza M.G., Santos E.M., Santos C.K.C., Silveira C.M., Santos S.H.S., de Paula A.M.B., Farias L.C., Guimarães A.L.S. (2019). Is HIF1-a deregulated in malignant salivary neoplasms?. Gene.

