# Supplementary figures and images for: Hypoxia-Induced Biosynthesis of the Extracellular Matrix Molecules, Perlecan and Fibronectin, Promotes the Growth of Pleomorphic Adenoma Cells In Vitro Models
Source: Biomedicines. 2023 Nov 6;11(11):2981. doi: 10.3390/biomedicines11112981 (PMC10669301; doi:10.3390/biomedicines11112981)

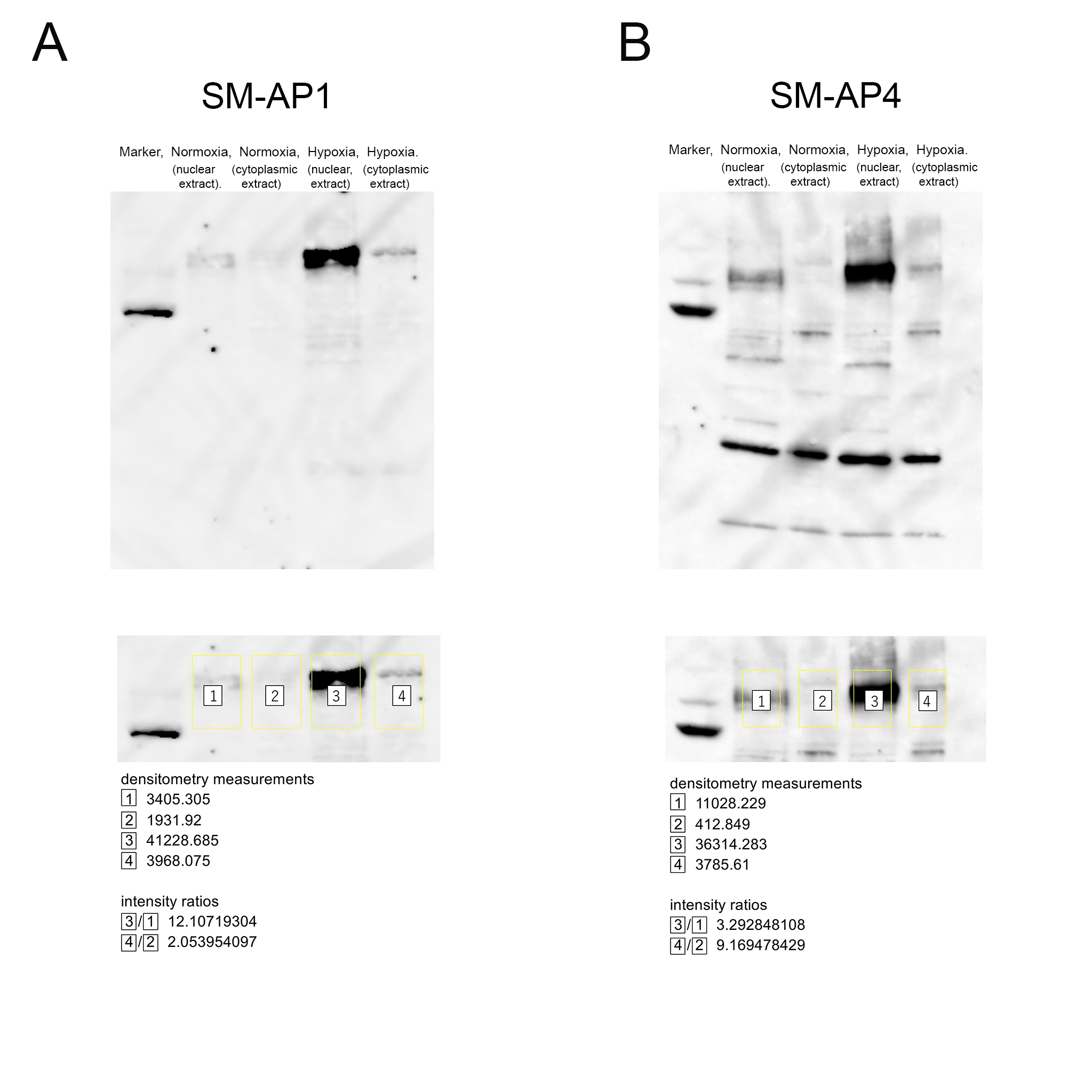

Supplement: Supplementary file 1 [file biomedicines-11-02981-s001.zip › Figure S1.tif]
